# Prevalence and pattern of lower extremity injuries due to road traffic crashes in Fako Division, Cameroon

**DOI:** 10.11604/pamj.2019.32.53.17514

**Published:** 2019-01-30

**Authors:** Palle John Ngunde, Asang Christian Ngwa Akongnwi, Chichom Alain Mefire, Fokam Puis, Eleanor Gounou, Ngwayu Claude Nkfusai, Udoamaka Glory Nwarie, Samuel Nambile Cumber

**Affiliations:** 1 Department of Medicine and surgery, Faculty of Health Sciences, University of Buea, Buea Cameroon; 2 Department of Microbiology and Parasitology, Faculty of Science, University of Buea, Buea, Cameroon; 3 Faculty of Health Sciences, University of the Free State, Bloemfontein, South Africa; 4 Section for Epidemiology and Social Medicine, Department of Public Health, Institute of Medicine (EPSO), The Sahlgrenska Academy at University of Gothenburg, Gothenburg, Sweden

**Keywords:** Prevalence, lower extremity injuries, Road traffic crashes, Fako Division

## Abstract

**Introduction:**

Low and middle income countries are disproportionately affected with road traffic injuries and the lower extremity is one of the most affected anatomical body parts. There exist very limited data on the pattern of lower extremity injuries in the Cameroon especially in the South West Region. We therefore, hypothesized that lower limb injuries are common in road traffic crashes and motorized two wheelers are the commonest cause.

**Methods:**

This was a hospital based prospective, cross sectional study. It involved four hospitals (Limbe and Buea Regional Hospitals, Baptist hospital Mutengene and Tiko District Hospital) in the Fako Division. It was carried out for three months. Victims of road traffic crashes received at emergency department of these hospitals during this period were assessed. Crash characteristics and injury characteristics were assessed and recorded.

**Results:**

We analyzed 411 crash victims, 197(47.93%) had lower extremity injuries. The male to female ratio was 1.4:1. Majority of crash victims were in their 3^rd^ and 4^th^ decades of life. The mean age of patients who had lower limb injuries was 33.30(±16.04). The most vulnerable road users were pedestrians (26.52%) and passengers on motor bikes (38.44%) and the commonest mechanism by which crash victims sustained injuries were: bike-car collisions (22.84%), and bike-pedestrian collisions (19.29%). Commercial motor bikes (62.77%) and taxis (22.38%) were the road users most involved in road traffic collisions. The leg 98(49.75%), thigh 23(11.68%), and knee 20(10.15%) were the most injured anatomical parts of the lower extremity. Fractures 68 (34.52%), lacerations 53(26.90%), and bruises 49(24.87%) were the most recurrent pattern of lower extremity injuries.

**Conclusion:**

In view of our findings we conclude therefore as follows: The prevalence of lower extremity injuries from Road Traffic Crashes in our study area was 47.93%. Associated risk factors to the road traffic crashes as identified by the victims were bad roads (10.15%) and bad weather (5.05%). The safety gargets were not adequately utilized by our victims, with 87.72% confirming that they did not wear the helmet and 87.50% affirming that they did not wear the seat belt at the time of the crash. The occupations mostly affected in our series were pupils and students (20.3%) and business people (19.2%), then the bike riders (15.23%). We thus recommend that the laws on the use of road safety gargets, especially helmets and seatbelts, be enforced, with riding and driving speeds reduced to below 60km/hour. Road usage should be avoided in bad weather and pedestrians lanes and zebra crossings be provided to minimize pedestrian-car or -bike collision.

## Introduction

The global burden of injury is on the rise [1-3] and Low and middle income countries have been revealed to be disproportionately affected. They account for about 90% of the overall injury related mortality [3]. Injuries would likely be the 4th leading cause of death by 2030 [4]. The Global Burden for Disease (GBD) predicts that road traffic accidents would rise to 6th place as a major cause of death by 2020, and would continue to rise if no additional road safety majors are put in place. A review study in low and middle income countries revealed that road traffic related injuries accounted for 30-86% of trauma related admissions [5]. In developing countries, motorbikes have emerged as a popular commercial mode of transportation without any corresponding improvement and adaptation of road infrastructures [6-8]. This is particularly true for Cameroon where road infrastructures are generally considered very poor. It has been estimated that the number of people who die as a result of traffic injuries in Cameroon is 35 times higher than what has been reported on similar road infrastructures in the United States of America. Vulnerable road users are most affected [9]. One third of traffic injury victims in large cities of Cameroon are motorcyclists [10]. Current data on injuries suggest that vulnerable road users, pedestrians, cyclist and motorized 2 and 3 wheelers constitute more than half (52%) of road users killed on the roads, with pedestrians alone constituting 37% [11]. The majority of persons killed on the roads are young adults, with 62% of all deaths aged between 15 and 44 years [11]. While in developed countries, effective intervention plans are implemented with measurable results [12-14], low and middle income countries still lack the most basic epidemiologic data. In Cameroon studies have been carried out generally on the effects of road traffic crashes in various regions and hospitals in Cameroon, but there exist very limited data on the pattern of lower extremity injuries in the country, especially in the South West Region of the country.

**Study objectives**: The objectives of this study were: to determine the prevalence of lower extremity injuries; To describe the demographic variables associated with lower extremity injuries; to identify types of lower extremity injuries (fractures, lacerations, contusions, bruises); to identify associated risk factors to Road Traffic Crashes and the use of safety measures by the victims; to identify the occupations most prone to get involved in Road Traffic Crashes.

## Methods

### Study design, study area and setting

It was a hospital based prospective cross-sectional study carried out in four major hospitals of the Fako Division. The South West Region is one of the 10 regions of Cameroon. It has a population of about 1.3 million according to the 2005 national census. It has 6 divisions (Fako, Koupe-Manengouba, Lebialem, Manyu, Meme, and Ndian). The Fako Division covers an area of 2093km2, and has a population of 534854 people. It includes five main towns; Limbe (capital), Buea, Mutengene, Tiko and Muyuka. The main means of transportation in the South West Region is by motor vehicles and motor bikes. The target population included patients with lower limb injuries obtained from road traffic crashes.

### Study population and sampling

The study involved every patient that was involved in a road traffic crash and presented with lower extremity injuries plus any associated injuries to the emergency department of the chosen hospitals (the Buea Regional Hospital, the Limbe Regional Hospital, Baptist Hospital Mutengene and the Tiko District Hospital) within the period of the study.

### Selection criteria

**Inclusion criteria:** Patients with injuries sustained from road traffic accidents during the period of the study. Patients with lower extremity injuries admitted in the hospital at start of the study that consented to participate in the study.

**Exclusion criteria:** Patients from whom relevant data could not be obtained. Victims of injuries secondary to other causes than road traffic crash, and patients who refused to consent to the study.

### Sample size

It was estimated using the Lorenz formula as seen below:

Where, n = minimum sample size required for this study; p = 53.6%; prevalence of lower extremity injuries following bike accidents in hospital based prospective cohort study in Laquintini hospital Douala.

d = precision= 0.05; z = coefficient of significance = 1.96

The sample size, n = (0.536) (0.464) (1.96) (1.96)) / (0.05) (0.05) = 382 Thus, the study was to include at least 382 participants.

### Study procedure

Patients who presented to the hospital with road traffic injuries were approached. Those who met the inclusion criteria were counselled on the aim and importance of the study. Those who granted consent were then examined and the data recorded on the data collection form. The data collection form had two main sections: the first section for the demographic variables and the second section for the crash and injury characteristics. We divided the days of the week to define week-days (from Monday to Thursday) and week-ends (from Friday to Sunday). We also divided the 24 hours of the day as day time (6 am to 5:59 pm) and night time (6 pm to 5:59 am). The data was collected simultaneously in all the four hospitals in their emergency departments. It went on for 3 months. Patients who arrived in a critical state were examined later in the ward when they were much improved and stable.

### Data collection, management and analysis

The data collected included: 1) patient’s identification: name, age, sex, level of education, injury characteristics, time of the accident. a) Type of crash: motor-bike or car; b) Type of collision: bike-bike, bike-car, car-car, pedestrian-bike, pedestrian-car; c) Position of the victim: pedestrian, bike rider or passenger, car driver or passenger; d) Body part injured and nature of the injury.

2) Crash characteristics: date and time of crash, type of collision, position of the patient (rider, driver or passenger), rider’s or driver’s experience (license), helmet or seat belt use and the notion of alcohol consumption by rider or driver within 6h prior to the crash.

3) Lesions identified and recorded: Description of these lesions included identification of anatomical location, detailed description of the lesion, and estimation of Glasgow Coma Score when indicated.

4) Outcome in the emergency department: discharge, Inward admission, referral to another institution, or death in the emergency department. The data collected using the data collection forms were thoroughly checked for discrepancies. Data recorded was checked again for completeness before data analysis.

Simple illustrations like graphs, tables, measures of central tendencies and dispersion were obtained for the variables. The chi square test for independence and the fisher’s test of significance were used where necessary to compare results. Epi Info software was used to analyse this data. Data was kept secret and all information was recorded in a computer protected by a password.

### Ethical consideration

Ethical clearance was obtained from the Faculty of Health Sciences Institutional Review Board. An authorization was sought from the Regional Delegate of Public Health of South West Region. Permission was obtained from the Directors of the Hospitals involved before the research was conducted. Participants received explanations on the goals of the research and why their collaboration was important.

## Results

A total of 434 road traffic crash victims were received across all 4 hospitals during the period of the study. Twenty-three (5.29%) patients were excluded. Out of these 6 did not consent, 16 had incomplete data and 1 was declared dead on arrival. Thus we finally had a total of 411 crash victims to analyze. The age ranged from 2 to 80 years, with a mean age of 32.6 (± 15.6) years. 95% CI 31.1-34.1. There were 268 males and 143 females, giving a male to female ratio of 1.87:1. There were 197 victims with lower limb injuries giving a prevalence of 47.93%.

### Demographic and social variables

**Figure 1:**
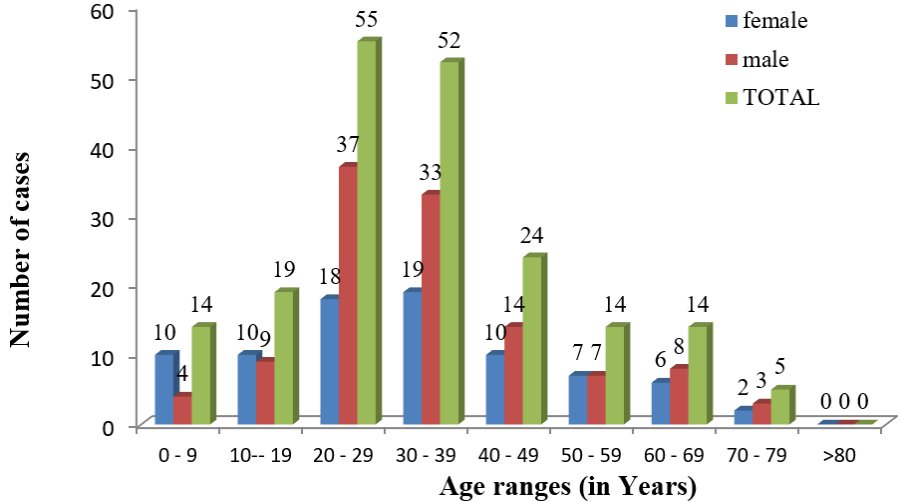
Gender distribution of lower extremity injuries per age group

Among the 197 patients that had lower limb injuries, 115 were males and 82 were females, giving a male to female ratio of 1.4:1. The mean age was 33.30(±16.04). The majority of patients (54.32%) were aged between 20 and < 40. Patients aged between 40 and < 60 were the second most affected group (19.29%), followed by children and teenagers (16.75%). Patients with lower limb injuries in the age group 0 < 20 were predominantly female, while the male gender predominated in the other age groups (Figure 1).

### Crash characteristics

**Figure 2:**
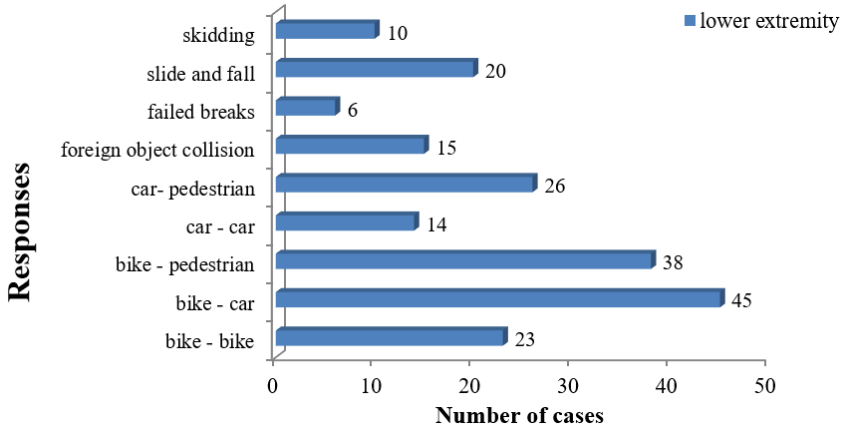
The mechanisms by which lower extremity injuries were obtained

The most common mechanism by which crash victims sustained lower limb injuries were: bike-car collisions (22.84%), and bike-pedestrian collision (19.29%). Thus motor bikes (76.14%) were the most common cause of lower extremity injuries (p < 0.01) (Figure 2).

Position of patients with lower limb injuries at the time of the crash

**Figure 3:**
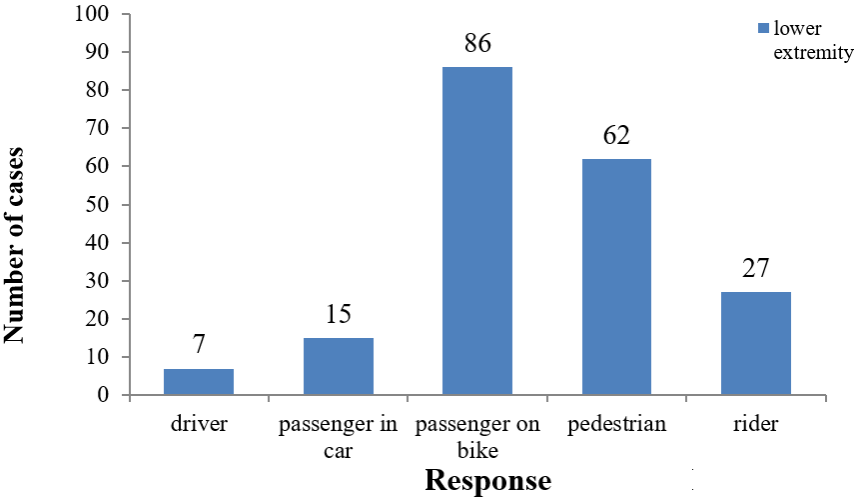
Position of patients with lower limb injuries at the time of the crash

The most vulnerable road users were passengers on motorized two wheelers (43.65%) and pedestrians (31.47%) (Figure 3).

### Most vulnerable road users per age group

Regarding the age groups, the three most vulnerable road users per age group are depicted in the table (in descending order) (Table 1). Regarding the day of the injury we noticed an increase in road traffic crash related injuries during festive periods or national day celebrations.

**Table 1:** Most vulnerable road users per age group

Age group (years)	Most vulnerable road users
**0-10**	Pedestrian Passenger on bike Passenger in car
**11-20**	Passenger on bike Pedestrian Passenger in car
**21-30**	Passenger on bike Passenger in car pedestrian
**31-40**	Passenger on bike Rider Passenger in car
**41-50**	Passenger on bike Pedestrian driver
**51-60**	Passenger on bike Pedestrian driver
**61-70**	Pedestrian Passenger on bike driver
**71-80**	Passenger on bike Pedestrian Passenger in car

### Road safety measures used

The road safety measures here were: helmet, seat belt, alcohol within 6 hours, drivers/ riders license. With regards to road traffic crash related risk factors, 20 (10.15%) admitted to bad roads as a related risk factor, while 10 (5.05%) pointed to bad weather as a related risk factor (Table 2).

**Table 2:** Road safety measures used

	Yes	No	Not applicable
**Helmet**	14(12.28%)	100(87.72%)	83
**Seat belt**	3(12.50%)	21(87.50%)	173
**Alcohol within 6 hrs.**	12(6.09%)	185(93.91%)	0
**Drivers/ Riders license**	11(36.67%)	19(63.33%)	167

### Pattern of lower extremity injuries

**Figure 4:**
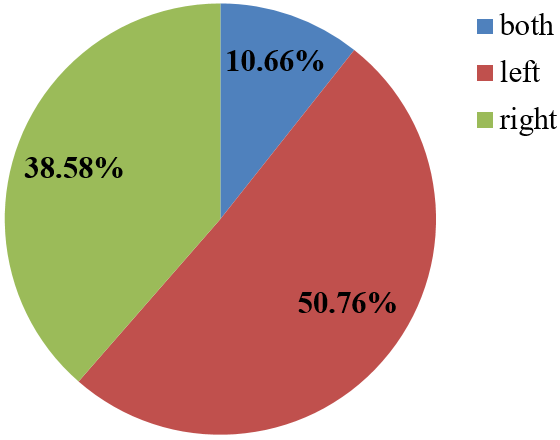
Side of lower limb affected

Regarding the side of the lower limb affected, the left lower limb 100 (50.76%) was generally more affected than the right 76 (38.58%). 21 (10.66%) crash victims had injuries on both lower limbs. This was true in both genders (Figure 4). The most injured anatomic regions of the lower limbs were: the leg 98 (49.75%), thigh 23 (11.68%), and knee 20 (10.15%). Out of the patients with lower limb injuries, 26 (13.20%) crash victims had injuries to multiple lower limb anatomic regions at the same time (Table 3).

**Table 3:** Frequency of injuries per anatomic region

Anatomical part of lower limb affected	Frequency	Percent
Ankle	16	8.12%
Foot	9	4.57%
Hip	5	2.54%
Knee	20	10.15%
Leg	98	49.75%
Multiple	26	13.20%
Thigh	23	11.68%
**TOTAL**	197	100.00%

### Anatomical part of lower limb affected per gender

Females had more ankle injuries 9(56.25%) and hip 3(60.00%) than males 7(43.75%) and 2(40.00%) respectively. As depicted in the table below, male crash victims dominated in all the other anatomic regions of the lower limb (Table 4). The most common pattern of lower limb injuries were Fractures 68 (34.52%), lacerations 53(26.90%), and bruises 49(24.87%). Most patients were conscious on arrival with > 95% of patients having a Glasgow coma score (GCS) of > 13 (Table 5). Regarding gender there were more fractures in males 44(64.71%) than females 24 (36.26%), (p value = 0.2).

**Table 4:** Anatomical part of lower limb affected per gender

Anatomic part/Gender	Ankle	Foot	Hip	Knee	Leg	Multiple	Thigh	TOTAL	P value
Female	9	2	3	9	40	9	10	82	0.005
Male	7	7	2	11	58	17	13	115	0.005
**TOTAL**	16	9	5	20	98	26	23	197	

**Table 5:** Pattern of injury

Pattern of lower extremity injury	Frequency	Percent
Amputation	1	0.51%
Bruise/ contusion	49	24.87%
Dislocation/ Subluxation	5	2.54%
Fracture	68	34.52%
Laceration/ wound	53	26.90%
Multiple	8	4.06%
Pain only	11	5.58%
Sprain/strain	2	1.02%
**TOTAL**	197	100.00%

### Types of fractures

**Figure 5:**
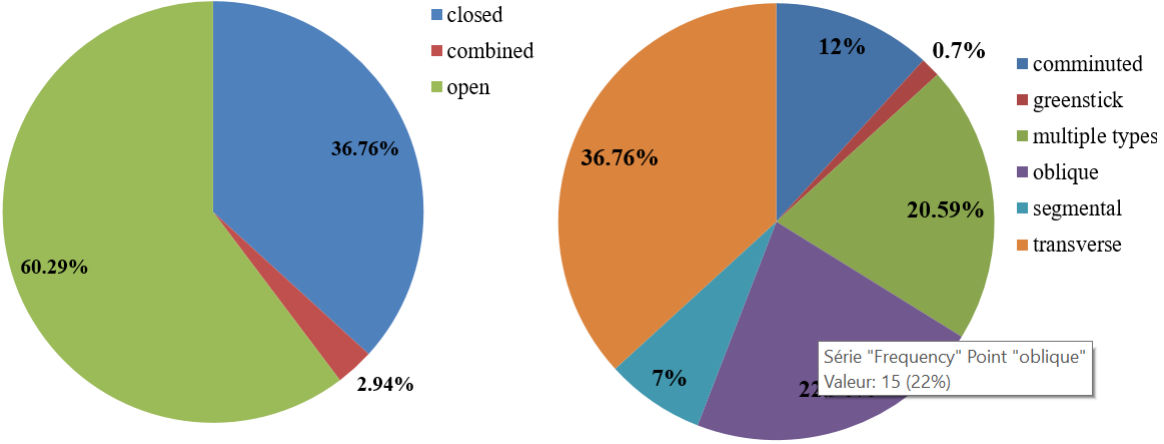
Types of fractures

Open fractures 41(60.29%) were more common than closed fractures 25(36.76%). Two crash victims (2.94%) had both types. Transverse fractures 25 (36.76%) and oblique fractures 15 (22.06%) were the two most common types of fracture observed. 14 (20.59%) of crash victims with lower limb fractures had multiple types of fractures (Figure 5).

**Bones affected by fractures:** Looking at the bones affected, the tibia 19(27.94%) and femur 19(27.94%) were most affected, 17(25.00%) crash victims had a combined tibio-fibular fracture.

**Figure 6:**
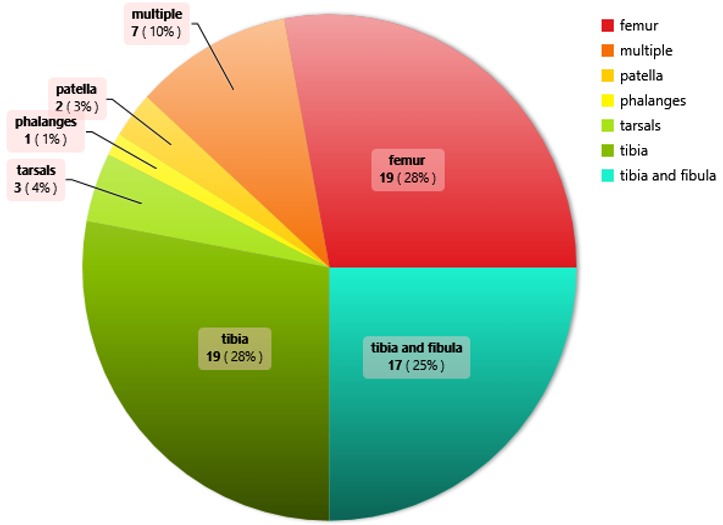
Bones affected by fractures

**Speed at which crash occurred:** Regarding the reported speed of the transporter involved, fractures were most common at speeds above 60km/hr. 43(63.24%) (Figure 6).

## Discussion

Reports on epidemiological studies on lower limb injuries due to road traffic crashes are scarce. This study found a prevalence of lower extremity injuries in road traffic crashes of 47.93%. This high prevalence is similar to that found in another study in Cameroon [15]. This high prevalence can be attributed to the fact that the lower extremities are most often very exposed compared to other anatomical parts of the body.

From this study, road traffic crash victims with lower extremity injuries can be divided into 3 groups with varying gender distribution. First are the children and teenagers (0 < 20) dominated by females, the young adults (20 < 50) the most affected age group and mainly dominated by males and lastly the senior citizens (> 50) with an almost equal gender distribution. In general, there was a marked male predominance in our study. Males are by their nature more active and so more likely to get them injured than females [16-18]. Lower limb injuries were most encountered (54.32%) in people in the active and productive age group (20 < 40) which correlates with other studies [16-22]. This is so because this age group comprises the active population and the main source of revenue and work force of our study area. They are more mobile, always moving between different locations by varied means of road transport and thus are most at risk of road traffic accidents. Motorized two wheelers (68.37%) were the most involved mode of transportation in road crash collisions. Motorized two wheelers are almost becoming the main means of transportation in most Cameroon cities and rural areas. Little formal training of the riders on how to operate the motorcycles and disregard for traffic regulations constitute a major risk factor for accidents [23].

Passengers on motor bikes (44%) and pedestrians (31%) were the most vulnerable road users affected with lower limb injuries. This is contrary to a study carried out in Nigeria by [24] in which passengers in minibuses and drivers were most vulnerable [24]. This is attributed to the fact that motorized two wheelers are the main means of intra urban transportation in most Cameroon cities. This large proportion of vulnerable road users is also explained by a traffic mix of incompatible users (pedestrians, cyclists, motorbikes, cars, and trucks) with, for example, communities living close to major highways, or the lack of pedestrian lanes along these highways [25].

Most previous studies describing the pattern of injuries in road traffic crash victims indicate a dominance of facial, extremity and head injuries [13, 26, 27]. This is similar to the findings we had with a marked high prevalence of head and lower extremity injuries. A very small percentage of these road users use the safety measures like helmets seat belts and little or no protective gear for the extremities. Decrease in the rate of use of protective measures reflects a lack of awareness of the role of these measures in preventing and reducing the severity of injuries.

There was a variation regarding the limb involved, the left limb (50.76%) was more affected than the right limb (38.58%, p > 0.05). A few patients sustained injuries on both limbs (10.66%). This was similar to findings by other researchers, although this was not statistically significant [28, 29]. In our study area, drivers and riders keep right, thus exposing the left half of the body to collisions with opposite flow traffic.

Fractures (34.52%) were the most common pattern of lower extremity injury sustained by road crash victims in this study, closely followed by lacerations (36.90%) and bruises (24.84%) [28, 29].This is similar to other findings [24], but different from other studies in which bruises and abrasions were the most common [13, 29].

### Study limitations

**Self-reporting:** The accuracy of respondent's answer on the occurrence of injury events or on how injuries were acquired could not be independently verified.

**Language difficulty:** Not all patients were fluent in English or French; this made it difficult for them to describe crash events in understandable manner. This might have affected our data collection. To fix this, we advised patients to use the language they were most fluent in and translators were sought in exceptional cases.

**Study type:** Being a hospital based study implied there was bound to be some under reporting of injuries as some of these patients would prefer to be treated locally or elsewhere. To reduce this, we had to select hospitals from all the stages of referral system. This will reduce under reporting and provide a clearer pattern of injury patterns, regardless of economic background and location.

**Consent issues:** Not all patients were willing to give consent. Others had incomplete data while some died at the crash scene. All these contributed to some degree of under reporting.

## Conclusion

From our findings and in view of our study objectives, we come to the following conclusions: The prevalence of lower extremity injuries from Road Traffic Crashes in our study area was 47.93%. More males sustained lower extremity injuries than females, with a male to female ratio of 1.4:1, and the majority of the victims in our series were between the ages of 20 to 40 years old. In our series the left lower limb was more affected than the right and fractures were the most common type of injuries sustained (34.52%), followed by lacerations (26.90%),and bruises (24.87%). Associated risk factors to the road traffic crashes as identified by the victims were bad roads (10.15%) and bad weather (5.05%). The safety gargets were not adequately utilized by our victims, with 87.72% confirming that they did not wear the helmet and 87.50% affirming that they did not wear the seat belt at the time of the crash. The occupations mostly affected in our series were pupils and students (20.3%) and business people (19.2%), then the bike riders (15.23%).

**Our recommendation:** Considering our findings we recommend the following to the road safety authorities and the general road user population: 1) That the laws on the use of road safety gargets while riding or driving, especially helmets or seat belts, should be enforced. 2) Road users should ride or drive below 60km/hour, especially when on bad roads. 3) Road users should avoid riding or driving in bad weather. 4) Pedestrian’s lanes and zebra crossings should be provided on the roads to minimize pedestrian-car or pedestrian-bike collision.

### What is known about this topic

- Road Traffic Crashes are a major problem in our study area, resulting from such factors as irrational road use from drivers and riders, poor road infrastructure, and non-respect of road rules;
- The increase in motorcycle use increases the rate of the accidents and leads to more limb injuries;
- Also previous studies show that more males are victims to road crashes than females.

### What this study adds

- We found out that most road traffic crashes involve mainly motorized two wheelers with the most vulnerable road users being pedestrians and passengers on motor bikes. Pupils, students and business pedestrians are more at risk of road traffic crashes;
- In the 20-30 years and the 30-40 years age groups, passengers on motorbikes were more at risk; the victims in the 0-10 year’s age group were mostly Nursery and primary school children who were going to or coming from school unaccompanied: the victims in the 20-30 and 30-40 years age groups were mostly young adults who used mostly commercial motorbikes for their transportation;
- In our series the left lower limb was more affected than the right and fractures were the most common type of injuries sustained (34.52%), followed by lacerations (26.90%), and bruises (24.87%): associated risk factors to the road traffic crashes as identified by the victims were bad roads (10.15%) and bad weather (5.05%); the safety gargets were not adequately utilized by our victims, with 87.72% confirming that they did not wear the helmet and 87.50% affirming that they did not wear the seat belt at the time of the crash.

## Competing interests

The authors declare no competing interests.
